# Evaluation of Solid and Hollow Sand Brick Properties with Partial Replacement of Fine Aggregates by Ground Granulated Blast Furnace Slag

**DOI:** 10.3390/ma19061250

**Published:** 2026-03-21

**Authors:** Kamal Hosen, Alina Bărbulescu

**Affiliations:** 1 Department of Mechanics, Materials and Structures, Budapest University of Technology and Economics, 1111 Budapest, Hungary; kamalhosen@edu.bme.hu; 2 Department of Civil Engineering, Transilvania University of Brasov, 5 Turnului Str., 500152 Brașov, Romania

**Keywords:** ground granulated blast furnace slag, sand bricks, sustainable construction, compressive strength, water absorption, thermal conductivity, industrial waste utilization, environmental impact

## Abstract

Ground granulated blast furnace slag (GGBFS), an industrial by-product of steel manufacturing, can be utilized as a partial replacement for natural fine aggregate in clay brick production. Although widely used in cementitious systems, its incorporation into sand bricks and its effects on key performance parameters remain insufficiently investigated. To fill in the gap, sand bricks containing 0–35% GGBFS (at 5% intervals) were tested for compressive strength, water absorption, thermal conductivity, and efflorescence. Optimal performance was achieved at 25% replacement. Compressive strength increased from 17.5 MPa (control) to 24 MPa (28.5% improvement). Water absorption decreased from 11.67% to 8.20% (29.7% reduction), and thermal conductivity decreased from 1.08 to 0.85 W/m·°C. No efflorescence was observed at 25% GGBFS, whereas higher replacement levels (30% and 35%) exhibited increased efflorescence. The results confirm that 25% GGBFS replacement enhances mechanical and durability-related properties of clay bricks, demonstrating its technical feasibility as an alternative fine aggregate.

## 1. Introduction

The construction sector in developing regions has expanded rapidly due to urbanization and rural development, resulting in a substantial increase in brick production demand [1,2,3,4]. Conventional kiln-fired clay brick manufacturing is environmentally unsustainable, involving extensive depletion of agricultural topsoil and river sand [5,6,7]. Moreover, brick firing predominantly relies on coal combustion [8,9], a major source of greenhouse gas (GHG) emissions and atmospheric pollutants [10,11].

In Bangladesh, 7881 registered traditional brick kilns operate nationwide [12], significantly degrading air quality in major urban centers such as Dhaka, Sylhet, Rajshahi, and Chattogram [4,13,14]. These kilns emit considerable quantities of CO_2_ and particulate matter (PM_2.5_ and PM_10_), with regional emissions estimated at 22 kt and 37 kt, respectively [15,16,17,18]. Such emissions exceed international health standards and are strongly associated with respiratory and cardiovascular diseases [19,20,21]. These environmental and public health concerns necessitate the development of low-carbon, resource-efficient masonry alternatives.

Ground granulated blast furnace slag (GGBFS), a glassy, granular byproduct of iron manufacture, offers a possible solution [22,23]. With a production ratio of approximately 300 kg of slag per metric ton of steel, GGBFS represents an abundant precursor for cementitious applications [24].

Zhao et al. [25] investigated the long-term hydration and microstructural evolution of blended cement containing GGBFS and waste Clay Brick Powder (CBP). Their study showed that GGBFS enhances early-age compressive strength, particularly within the first three months, by accelerating hydration and consuming more portlandite, resulting in a denser microstructure. In contrast, CBP initially reduces compressive strength due to its slower reactivity but contributes to strength gain over time by refining larger pores into smaller diameters. Both materials improve porosity control, with GGBFS reducing pore sizes below 50 nm and CBP mitigating larger pores through gel formation. These findings highlight the potential of GGBFS and CBP to optimize mechanical performance and durability through microstructural modification, suggesting that similar benefits may be achievable when GGBFS is incorporated into fired clay bricks.

Current research emphasizes the mechanical and microstructural benefits of incorporating slag into various brick matrices. Surul et al. [26] demonstrated that as firing temperatures increased from 850 °C to 1050 °C, GGBFS-clay composites exhibited reduced porosity and enhanced mechanical strength, concluding that a 40% substitution rate yields performance metrics comparable to traditional units.

Zawrah et al. [27] investigated the synthesis of geopolymers using GGBFS and fired clay “grog.” Their findings revealed that a 60% slag/40% clay mix achieved a superior compressive strength of 83 MPa after a 90-day curing period, attributed to the densification of the geopolymeric matrix and a significant reduction in water absorption. Yu et al. [28] synthesized composite bricks by incorporating municipal sludge (MS) and blast furnace slag (BFS) into kaolin clay (KC). The optimal compressive (15 MPa) and flexural (38 MPa) strengths were achieved using 10% BFS and 40% MS. They found that BFS promoted liquid glassy phase formation, enhancing brick performance. Kumar et al. [29] shifted focus toward alkaline activation, utilizing sodium silicate and hydroxide to trigger the aluminosilicate reaction in geopolymer blocks containing iron ore tailings and slag sand, noting that the liquid-to-binder ratio is critical for strength gain that improved over 7, 14, and 28 days.

Dom et al. [30] evaluated GGBFS as a partial replacement for Ordinary Portland Cement (OPC) in sand-cement bricks. Their longitudinal study identified a 20% replacement threshold as optimal, yielding a maximum compressive strength of 60.9 MPa at 56 days and minimal water absorption (3.11%) over 90 days.

Hashim et al. [31] explored whether using GGBFS instead of OPC would be more sustainable when producing sand-cement bricks. For this aim, they tested three different percentages of GGBFS in the mix—30%, 40%, and 50%—and looked at the physical and mechanical properties of the sand-cement bricks, including water absorption and compressive strength, after a curing time of both 7 days and 28 days. The results indicated a strong correlation between the percentage of GGBFS in the mix and the resultant compressive strength. Specifically, bricks produced with 30% GGBFS exhibited the highest compressive strength after 28 days (47.6 MPa) and had lower water absorption than bricks produced with less than 30% GGBFS. Lopez-Perales et al. [32] studied the partial substitution of flint clay in refractory castables with BFS to improve thermal conductivity and other physical properties of the castable. The authors prepared castables that contained varying amounts of BFS (0–20% by wt.) instead of flint clay, and all samples were fired up to 1400 °C. Their findings indicated that 10% by wt. of BFS resulted in more anorthite, a decrease in the porosity, and an increase in the density and thermal resistance of the refractories. However, the addition of 15–20% by wt. BFS caused volumetric increases due to the formation of cristobalite.

Arya and Vanreyk [33] investigated the benefits of adding different percentages (4%, 8%, 12%, and 16%) of GGBFS to clay brick. The maximum compressive strength (10.13 N/mm^2^) occurred at 8% GGBFS. A direct correlation was observed between GGBFS concentration and water absorption capacity. As the GGBFS content reached a 16% substitution threshold, water absorption peaked at 20.08%. Despite this increased porosity, the specimens exhibited negligible efflorescence, suggesting a low concentration of soluble alkaline salts within the clay–slag matrix. This chemical stability is vital for long-term aesthetic and structural durability in saline or humid environments. The introduction of GGBFS serves as a thermal modifier. At a 16% concentration, the thermal conductivity was reduced to 0.86 W/m °C. This decrease in conductivity enhances the material’s thermal resistance, contributing to superior building envelope insulation and energy efficiency. However, mechanical performance exhibited an early peak; compressive strength reached its maximum of 4.9 N/mm^2^ at a 5% substitution rate, beyond which strength gains diminished.

The research conducted by Divahar et al. [34] validates the broader industrial utility of these composite materials, demonstrating that the integration of GGBFS facilitates an energy consumption reduction of up to 30% throughout the manufacturing lifecycle. This attenuation in embodied energy is primarily attributed to the reduction in thermal requirements during the firing phase (or the transition to ambient-temperature curing). By mitigating the carbon intensity and resource demands of production, this approach significantly lowers the environmental footprint while simultaneously reducing the marginal unit cost.

Beyond GGBFS, interest has grown in other sustainable materials for bricks. Chen et al. [35] studied compressed mud bricks incorporating stone dust, wheat straw, and cement, showing that these additives significantly increased compressive strength and the modulus of elasticity. These results suggest that GGBFS-modified bricks could similarly benefit from incorporating industrial by-products, enhancing mechanical performance and durability while reducing the carbon footprint associated with conventional brick production.

Ansari and Kumar [36] investigated how to create sustainable geopolymer bricks from GGBFS and fly ash in conjunction with their studies on using cementitious applications of GGBFS. Their work indicated that the compressive strength could be increased for both types of brick with thermal curing and alkaline activation, providing solutions for using GGBFS as a supplementary binder in both conventional and geopolymeric types of bricks. Although their focus was primarily on the chemical activation of GGBFS, the current research focuses on partially replacing aggregates with GGBFS in traditional bricks. This alternative method provides a more energy-efficient, scalable way to improve the properties of bricks while supporting sustainable construction methods. Moreover, these objectives can be achieved with little to no additional chemical processing or special curing conditions.

Faraji et al. [37] produced geopolymer foam concrete using GGBFS, waste concrete sludge (WCS), and waste bamboo powder (WBP), activated with sodium metasilicate. GGBFS enhanced geopolymerization due to its amorphous structure, improving strength, insulation, and durability. The optimal mix (0% WCS, 5% WBP) achieved a compressive strength of 11.4 MPa at 28 days with high freeze–thaw resistance. Incorporating GGBFS and waste materials reduced the environmental impact, water demand, shrinkage, and water absorption.

Zhang et al. [38] evaluated graphene oxide (GO) in a ternary geopolymer system (fly ash, GGBFS, and steel slag) using Joule heating curing (JHC). With 0.03 wt% GO and curing at 15 V for 8 h, compressive and flexural strengths reached 50.47 MPa and 4.66 MPa, respectively, exceeding conventional oven curing results. GO improved the microstructure by promoting N(C)-A-S-H gel formation, reducing porosity, and enhancing electrical conductivity.

Wang et al. [39] examined geopolymer grout made from circulating fluidized bed combustion (CFB) fly ash and GGBFS for highway subgrade stabilization. Up to 70% CFB met flow, setting, and strength requirements. GGBFS enhanced reactivity, early strength, and freeze–thaw resistance. The optimal mix (65–70% CFB, 30–35% GGBFS) demonstrated micro-expansive behavior beneficial for subgrade stabilization, confirming the potential of CFB-based geopolymers as sustainable and durable construction materials.

Recent studies have shown that curing conditions, most importantly temperature and humidity levels, play a vital role in affecting the hydration behavior, microstructure evolution, and mechanical properties of GGBFS-based cementitious materials [40,41]. Shumuye et al. [40] found that GGBFS cement composites cured at elevated temperatures (45 ± 2 °C and 95% RH) experienced fast hydration and a compacted hydrate phase compared to similar samples cured at lower temperatures, which increased early-age compressive strength and decreased microstructure porosity, although lower temperature samples are better suited for long-term structural stability than those cured at higher temperatures. Similarly Elkhadiri et al. [41] found that elevated curing temperatures influence the type, structure and stability of hydration products—such as C-S-H gel—by promoting pozzolanic activity and refining pore structure for improved strength; however, extremely high temperatures may inhibit continued uniformity in later hydration processes due to increased porosity and microstructural irregularity as the degree of hydration continues to increase with time. This demonstrates the importance of controlling laboratory curing conditions (both temperature and relative humidity) to provide continual hydration of both the cement and GGBFS, to achieve optimal mechanical performance and durability when designing and producing long-term stable blended brick products.

Although most studies emphasize mechanical strength and durability to support sustainability objectives, dynamic behavior is equally critical for real-world structural applications. Mansour et al. [42] investigated the dynamic response of historical masonry structures, focusing on the Santa Teresa Masonry Bridge in Italy. Their work demonstrated how masonry systems can be structurally characterized under dynamic loading conditions, providing insights relevant to seismic assessment and rehabilitation. Such findings underline the importance of evaluating not only the strength but also the dynamic stability of GGBFS.

Despite the growing body of literature on GGBFS, a significant knowledge gap remains regarding the high-resolution performance mapping of GGBFS in hand-molded masonry units. Most existing studies rely on broad substitution intervals and deterministic averages, failing to account for the inherent variability of manual production or the long-term kinetics of latent hydration. This study addresses these gaps by evaluating the systemic integration of GGBFS into brick production through an extensive experimental program involving 800 specimens. The novelty of this research is defined by three primary contributions:

- High-Resolution Substitution Analysis: Unlike studies using 10% increments, this work employs a 5% incremental gradient (0–35%). This allows for the precise identification of the “inflection point” where GGBFS transitions from a beneficial micro-filler to a potential disruptor of the matrix.
- Longitudinal Durability Mapping: While most research concludes at 28 days, this study extends the characterization to 56 days. This captures the late-stage hydration kinetics of GGBFS, providing a detailed view of how moisture absorption, efflorescence, and thermal conductivity stabilize over time.
- Stochastic Reliability Framework: Shifting from laboratory “best-case” averages, this study introduces statistical characteristic values (*f_k_*). By analyzing the standard deviation and 5% fractiles, we establish a reliability framework that accounts for the variability of hand-molded manufacturing, providing field-ready metrics for sustainable construction.

The primary objective is to establish an optimized framework for GGBFS-modified bricks—both solid and hollow—that meet or surpass structural standards while replacing non-renewable raw materials with industrial by-products.

## 2. Methodology

### 2.1. Experimental Program

To conduct this study, 800 bricks were produced using eight different percentages (0%, 5%, 10%, 15%, 20%, 25%, and 35%) of GGBFS content, as presented in detail in Section 2.2. Each experiment was done on 5 solid and 5 hollow bricks, and the average values were reported.

The experimental program included:

(a) Mix design and brick production—Section 2.2.
(b) Curing periods of 7, 14, 28, and 56 days.
(c) Testing Procedures:
  ▪ Compressive strength test (Section 2.3.1): 320 bricks were used, i.e., (5 solid + 5 hollow bricks) × 8 GGBFS compositions × 4 curing periods.
  ▪ Water absorption test (Section 2.3.2): 320 bricks were used, i.e., (5 solid + 5 hollow bricks) × 8 GGBFS compositions × 4 curing periods.
  ▪ Efflorescence test (Section 2.3.3): 80 bricks were used for testing only after 56 days, i.e., (5 solid + 5 hollow bricks) × 8 GGBFS compositions.
  ▪ Thermal conductivity test (Section 2.3.4): 80 bricks were used for testing only after 56 days, i.e., (5 solid + 5 hollow bricks) × 8 GGBFS compositions.

### 2.2. Materials and Brick Production

#### 2.2.1. Materials

**Figure 1:**
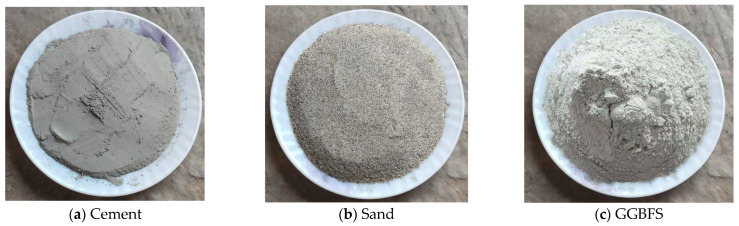
Photos of the materials used in this study: (**a**) cement, (**b**) sand, (**c**) GGBFS.

The following materials (Figure 1) have been used to conduct this research.

(d) Cement: Ordinary Portland Cement (OPC) was obtained from the local market in Bangladesh. The physical characteristics of the cement were assessed by BDS EN 197-1:2010 [43], which specifies the composition, specifications, and standards that are applied to common cement. The guidelines for the testing methodology outlined within this standard were adhered to throughout the testing, and the results of each of the tests are found within Table 1. BDS EN 197-1:2010 is a widely accepted standard within Bangladesh for assessing the quality of cement against the internationally recognized standard (EN 197-1:2011 [44]).
(e) GGBFS: It was sourced from the local market in Bangladesh. The physical properties of GGBFS are provided in Table 2.
(f) Sand: It was sourced from the local market in Bangladesh. The physical properties of the sand are provided in Table 3.

#### 2.2.2. Mix Proportion and Casting Process

The weight of each component was used to determine the mix proportions. Eight design mixes were used (Table 4). The cement proportion remained constant for all design mixes to evaluate how the amount of GGBFS in the brick affects its performance. The mixing of the raw materials was conducted using a consistent water/cement ratio of 0.52 and an even mixing method, ensuring the mixture was always uniform and easily controllable throughout the entire process. GGBFS serves a twofold purpose as both a filler and a latent hydraulic binder.

When GGBFS is cured in the presence of water, the reaction between GGBFS and calcium hydroxide released during the hydration of cement will create additional C-S-H phases, refining the pore structure of the concrete. These chemical interactions will improve the microstructure of the concrete by reducing capillary pores and increasing binding between the particles, thereby producing stronger bricks and decreasing the amount of water absorbed by the tested brick specimens [45].

Eight hundred solid and hollow bricks were cast. Both solid and hollow bricks had identical external dimensions of 254 mm in length, 127 mm in width, and 76.2 mm in height. The hollow bricks featured a centrally positioned hole with a diameter of 25.4 mm running through the middle.

The raw material mixture was placed into wooden molds, and then, a manual compaction of the mixture took place by creating 25 compact blows in three layers on top of each other to prevent the formation of voids in the compacted material and provide uniform densification of all the molds. After compactification, the upper surfaces of the specimens were leveled and smoothed using a trowel to achieve a flat finish. The specimens were then air-cured for 24 h to attain sufficient initial strength before being demolded for subsequent curing and testing.

#### 2.2.3. Water Curing

After demolding, the specimens were submerged in potable water and cured for periods of 7, 14, 28, and 56 days. This curing approach was adopted because the brick’s strength development is primarily governed by cement and GGBFS hydration rather than by thermal or kiln-firing processes. Prolonged water curing promotes continued hydration, enabling effective bonding of the constituent materials and resulting in higher compressive strength. During this process, water penetrates the porous structure of the bricks, allowing the hydrating compounds to fully react and form dense, solid aggregates. Consequently, water curing plays a critical role in ensuring the durability and structural integrity of the bricks.

Controlled conditions were implemented for curing the bricks in a laboratory setting for uniform hydration and consistent development of the differing properties of the material. After the bricks had been demolded, they were fully submerged in water for curing under standard conditions of 23 °C ± 2 °C and a relative humidity of 90% for the respective curing times of 7, 14, 28, and 56 days. These conditions represent those typically found in curing cement-based materials and are known to allow for optimal hydration of the cementitious material (GGBFS-modified bricks). The temperature and humidity were monitored periodically throughout the entire curing period to ensure the conditions were consistent, as these factors greatly affect the rate at which the cement and GGBFS components will hydrate and therefore the strength and water absorption (durability) of the completed bricks.

### 2.3. Testing the Brick Properties

At the end of each curing period, the bricks were tested for compressive strength, water absorption, efflorescence, and thermal conductivity, as they provide critical insights into the performance and durability of the bricks. All testing was carried out in a laboratory to ensure controlled conditions and adherence to standardized procedures, thereby ensuring the accuracy and reliability of the results.

#### 2.3.1. Compressive Strength Test

The compressive strength of the bricks was determined following EN 772-1:2011 [46]. After the specified curing period, the representative brick specimens were selected, cleaned, and their dimensions measured using a vernier caliper to calculate the loaded area. Specimens with uneven surfaces were capped with cement mortar to ensure uniform load distribution. Each brick was placed centrally between the plates of a calibrated compression testing machine, with the load applied perpendicular to the bed face. A gradually increasing load was applied at a constant rate until the specimen failed. The maximum load at failure was recorded, and the compressive strength was calculated as:

(1) $$\mathrm{Compressive}\mathrm{Strength}\;(\mathrm{MPa})=\frac{\mathrm{Maximum}\mathrm{Load}\;(N)}{\mathrm{Loaded}\mathrm{Area}\;({\mathrm{mm}}^{2})}$$

This test provides information on the structural capacity and durability of the bricks, ensuring their suitability for masonry construction.

#### 2.3.2. Water Absorption Test

Water absorption was measured in accordance with EN 772-1:2011—Methods of test for masonry units: determination of water absorption of clay and calcium silicate masonry units by cold water absorption [46]. The specimens were first dried in an oven at 105 ± 5 °C for 24 h to obtain their dry weight (*W*_1_). They were then fully submerged in water at room temperature for 24 h, after which the wet weight (*W*_2_) was recorded. The water absorption percentage was calculated using the formula:

(2) $$\mathrm{Water}\mathrm{Absorption}\;(\%)=\frac{W_{2}-W_{1}}{W_{1}}\times 100$$

This test evaluates the porosity of the bricks, which directly affects their durability and resistance to moisture penetration.

#### 2.3.3. Efflorescence Test

The efflorescence on the brick surface was assessed according to [47]. Each brick was partially immersed in water to a depth of 25 mm for 24 h and then allowed to dry at room temperature. The formation of white crystalline deposits on the surface was visually inspected and classified as: Nil (No visible efflorescence), Slight (Less than 10% surface coverage), Moderate (10–50% surface coverage), Heavy (more than 50% surface coverage). This test provides insight into the potential for salt migration and long-term durability issues in masonry structures.

#### 2.3.4. Thermal Conductivity Test

The thermal conductivity of the brick specimens was determined to evaluate their thermal insulation performance. The test was conducted according to ASTM C177-19 [47] and ASTM C518-15 [48].

After completion of the curing period, representative solid and hollow brick specimens were selected and dried in an oven at 105 ± 5 °C until a constant mass was achieved to eliminate the influence of moisture on thermal performance. The dimensions of each brick were accurately measured to determine the thickness and cross-sectional area required for calculation.

Each specimen was placed between a hot plate and a cold plate within the thermal conductivity testing apparatus. A controlled temperature gradient was applied across the specimen, allowing heat to flow steadily from the hot side to the cold side. The test continued until steady-state heat transfer conditions were achieved, as indicated by constant heat flow and stable temperature readings. The heat flux passing through the specimen and the corresponding temperature difference across its thickness were recorded.

Thermal conductivity was calculated using Fourier’s law of heat conduction:

(3) $$k=\frac{Q\times L}{A\times \Delta T}$$

where k = thermal conductivity (W/m·K), Q = steady-state heat flow rate (W), L = thickness of the specimen (m), A = cross-sectional area (m^2^), and ΔT = temperature difference across the specimen (*k*).

The thermal conductivity test provides a quantitative measure of the ability of bricks to conduct heat. Lower thermal conductivity indicates better thermal insulation performance, which is essential for improving the energy efficiency of building envelopes.

The results were used to compare the thermal performance of bricks with varying GGBFS contents and to assess the suitability of these bricks for sustainable construction.

### 2.4. Statistical Analysis

To ensure the reliability of the mechanical results and account for material variability, a stochastic approach was employed to calculate characteristic values. Following standard masonry testing protocols (e.g., Eurocode 6), the characteristic compressive strength ($f_{k}$) was determined, assuming a normal distribution of the experimental values. The calculation is defined by the following probabilistic relationship:

(4) $$f_{k}=f_{m}-j\times s$$

where

⮚ $f_{m}$ is the mean experimental value obtained from *n* = 5 samples.
⮚ *s* is the sample standard deviation, representing the dispersion of data.
⮚ *j* is the statistical coefficient, taken as 1.64 to define a 5% fractile at a 95% confidence level.

This probabilistic framework allows for the quantification of variability across different GGBFS percentages and brick types (solid and hollow), moving beyond deterministic averages to provide values suitable for structural safety assessments [49].

## 3. Results and Discussions

### 3.1. Results of Compressive Strength Test

At the end of each curing period (7, 14, 28, and 56 days), the compressive strength of five bricks from each design mix was measured and compared against the BDS brick standard, which classifies bricks into three grades based on compressive strength: Grade S (≥24 MPa), Grade A (≥15 MPa), and Grade B (≥10.3 MPa).

**Figure 2:**
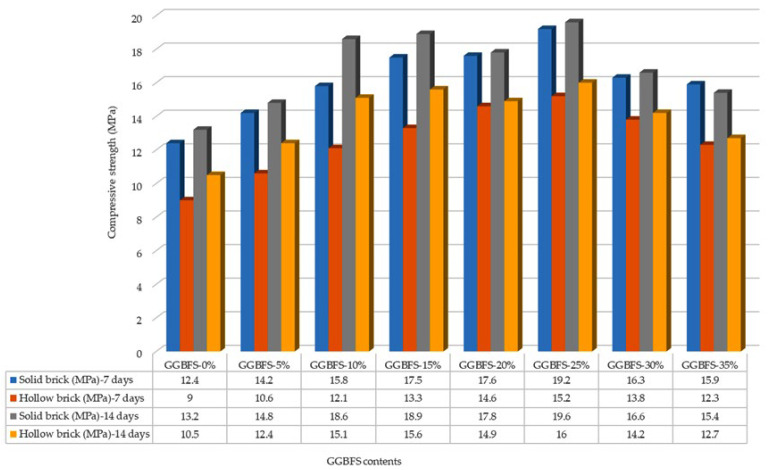
Compressive strength (in MPa) test results of bricks at 7 and 14 days of curing.

**Figure 3:**
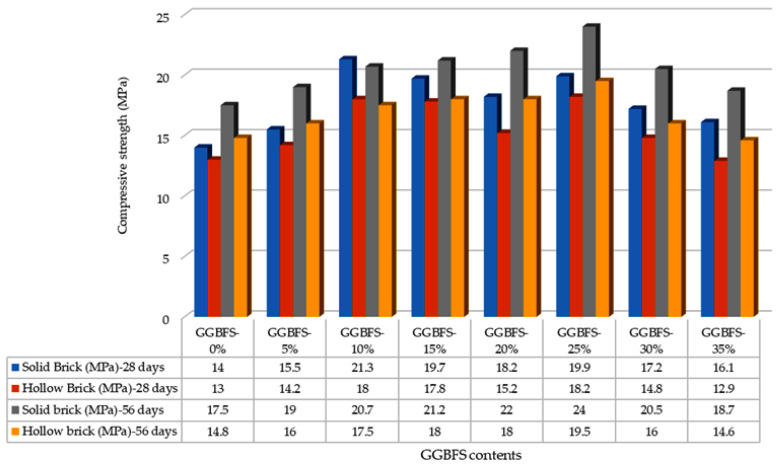
Compressive strength (in MPa) test results of bricks at 28- and 56-day curing.

Figure 2 shows the compressive strength test results at 7 and 14 days of curing period. For solid bricks, the 7-day curing tests show that the brick with 25% GGBFS achieved the highest compressive strength of 19.2 MPa, corresponding to Grade A. At 14 days, the brick with 25% GGBFS again recorded the highest strength of 19.6 MPa, also falling within Grade A. Figure 3 shows the compressive strength test results at 28 and 56 days of curing period. By the 28-day curing period, the highest compressive strength was observed in the brick containing 10% GGBFS at 21.3 MPa (Grade A), while the lowest strength was recorded in the 35% GGBFS brick at 16.1 MPa (Grade B).

At 56 days, the brick containing 25% GGBFS achieved the maximum compressive strength of 24.0 MPa, meeting the Grade S requirement. The two lowest-performing bricks at this stage were the 35% GGBFS (18.7 MPa) and 0% GGBFS (17.5 MPa) bricks, both meeting the Grade B requirement. Throughout all curing periods, solid bricks containing 25% GGBFS consistently exhibited the highest compressive strength, ultimately reaching Grade S after 56 days. For hollow bricks, the effect of GGBFS content was slightly different. At 7 days, increasing the GGBFS content initially increased compressive strength; however, a decline was observed in bricks with 30% (13.8 MPa) and 35% GGBFS (12.3 MPa).

The optimal performance defined by GGBFS at 25% is due to the pozzolanic reaction that takes place between the GGBFS and the calcium hydroxide released during cement hydration. As a result of this pozzolanic reaction, additional CSH is generated, which fills the capillary voids, refines the microstructure, and improves the ladder-like bonding between particles, resulting in improved compressive strength and reduced water absorption. However, as the GGBFS content increases above 30%, the performance begins to decline due to the dilution effect of the cement, where the relative amount of cement is insufficient to the facilitate full reaction of the GGBFS, leading to decreased levels of hydration products, incomplete filling of capillary voids, and increased microstructural irregularities, all of which contribute to a reduction in mechanical strength and durability of the bricks; hence this microscopic explanation is consistent with the trends observed in both solid and hollow bricks.

After 14 days, compressive strength increased for all percentages of GGBFS. After the 56-day curing period, the hollow brick containing 25% GGBFS outperformed all other mixes with a compressive strength of 16.0 MPa. This confirms that the curing period has a significant impact on the compressive strength of hollow bricks. The findings of this study are consistent with those reported by Surul et al. [26], who investigated bricks produced using GGBFS and flying ash. They observed that after 56 days of curing, the bricks with 25% GGBFS attained a maximum compressive strength of 24.0 MPa (Grade S). However, increasing the GGBFS content to 30% and 35% resulted in reduced compressive strength, corresponding to Grade B (≤15 MPa). Both studies highlight that compressive strength improves with extended curing periods, emphasizing the critical importance of adequate curing to achieve the optimal performance of manufactured bricks.

Similarly, Dom et al. [30] reported comparable trends in sand-cement bricks containing GGBFS. Both studies [26,30] concluded that moderate GGBFS contents, ranging from 10% to 25%, yielded the highest compressive strength, with 25% GGBFS producing the maximum values. Here, solid bricks containing 25% GGBFS reached 24.0 MPa (Grade S) after 56 days of curing, while hollow bricks with the same GGBFS content achieved 16.0 MPa (Grade A). By contrast, in [30], a compressive strength of 60.9 MPa at 56 days was reported when using 20% GGBFS.

The results from [26,30] also show that higher GGBFS percentages (30–60%) initially reduced early-age strength, although compressive strength increased over time. In the current research, bricks with 30% and 35% GGBFS consistently remained within Grade B (≥10.3 MPa) across all curing periods, reflecting trends like those observed in [30].

Hashim et al. [31] reported that 30% GGBFS yielded the highest compressive strength of 47.6 MPa at both the 28-day and 56-day tests, significantly higher than the results obtained in this research. While the studies [26,30] concluded that moderate GGBFS (25% to 30%) improved the compressive strength, Hashim et al. [31] concluded that the compressive strength of bricks with 30% GGBFS consistently outperformed that of the bricks with 25% GGBFS content during the initial stages of curing. In contrast, our results show that 25% GGBFS is the optimal content, particularly for long-term curing periods of 56 days, where it achieves the highest compressive strength for both solid and hollow bricks. This highlights that while slightly higher GGBFS contents may improve early strength, moderate replacement ensures the best long-term structural performance.

### 3.2. Results of Water Absorption Test 

**Figure 4:**
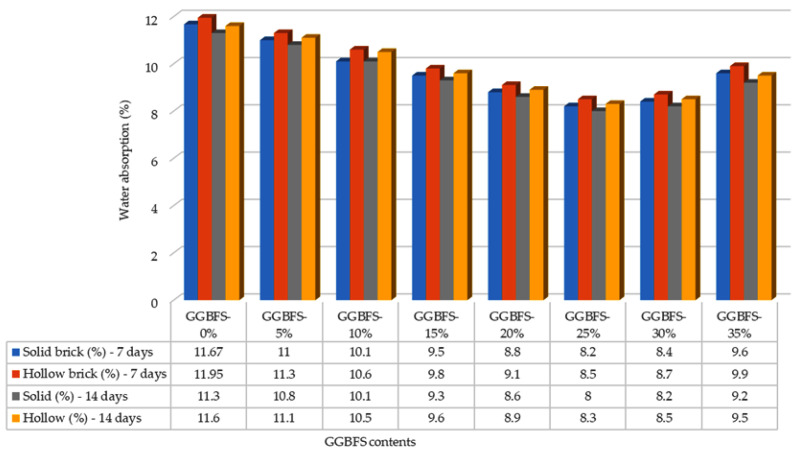
Absorption (in %) test results of bricks at 7 and 14 days of curing.

At the end of each curing period (7, 14, 28, and 56 days), the water absorption capacities of five bricks from each design mix were measured and compared with the BDS brick standard, which classify bricks according to their water absorption performance. Based on these standards, bricks are categorized into three grades: S-class (≤10%), A-class (≤15%), and B-class (≤20%), depending on the percentage of water absorbed. Figure 4 shows the water absorption test results at 7 and 14 days of curing period. After 7 days, for bricks containing 0% GGBFS, the average water absorption was 11.67% for solid bricks and approximately 11.95% for hollow bricks, placing both within the A-class category according to BDS requirements.

**Figure 5:**
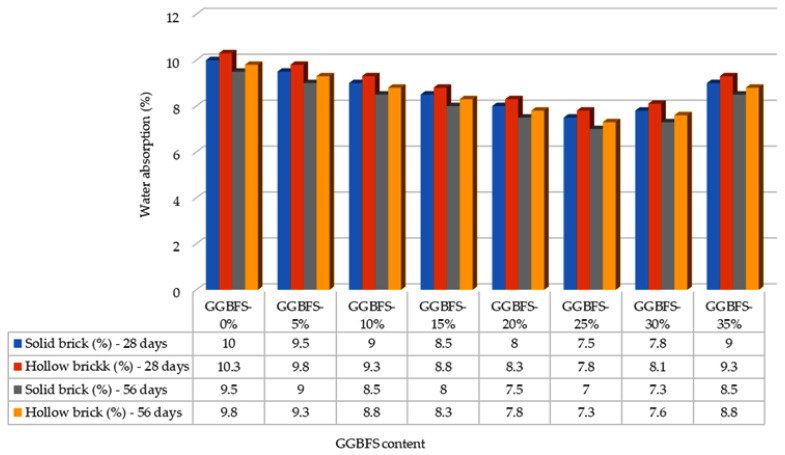
Absorption (in %) test results of bricks at 28 days and 56 days of curing.

As the GGBFS content increased, variations in water absorption were observed. At 5% GGBFS, solid bricks exhibited an average absorption of 11.00%, while hollow bricks absorbed approximately 11.30%. At 10% GGBFS, water absorption further decreased to 10.10% for solid bricks and 10.60% for hollow bricks. After 14 days of curing, the average water absorption capacity of solid bricks and hollow bricks were 11.30% and 11.60%, respectively. Figure 5 shows the water absorption test results at 28 and 56 days of curing period.

With continued curing and increasing GGBFS content, a further reduction in water absorption was observed between 28 and 56 days. By the end of the 56-day curing period, solid bricks with 0% GGBFS recorded an average absorption of 10.00%, while hollow bricks recorded 10.30%. These results indicate that both GGBFS incorporation and extended curing periods contribute to a reduction in water absorption for solid and hollow bricks. The most significant decrease in water absorption occurred between 7 and 14 days of curing, demonstrating that as hydration progresses and GGBFS content increases, pore volume within the bricks is reduced. This densification of the microstructure leads to enhanced resistance to water penetration and improved durability.

The environment where curing occurs is essential in the process of hydration for both cement and GGBFS, which have a major effect on the resulting properties (i.e., compressive strength, water resistance, etc.) of the manufactured bricks. The temperature and humidity conditions throughout the curing process dictate the rate at which both the cement and GGBFS fragment hydrating materials (C-S-H gel) are produced. Controlled curing conditions (23 °C ± 2 °C and 90% relative humidity) allowed complete hydration to occur for both cement and GGBFS, resulting in the superior mechanical performance (compressive strength and water resistance) of the bricks. The extended curing period (up to 56 days) allowed for additional hydration and development of the microstructure and enabled the bricks to reach their maximum compressive strength and optimal performance capabilities. Variances in the curing conditions (i.e., erratic temperature, low humidity) can cause incomplete hydration of cement and GGBFS and decrease the performance characteristics of the material.

The results of the water absorption tests obtained in this study are in good agreement with the findings reported by Surul et al. [26], particularly with respect to the overall trend observed. Both studies indicate that the incorporation of GGBFS into the brick mixture leads to a reduction in water absorption for both solid and hollow bricks, as the addition of GGBFS contributes to the formation of denser and heavier brick units. In this study, water absorption decreased as the GGBFS content increased up to 25%, with solid bricks showing a reduction from 11.67% to 8.20% and hollow bricks from 11.95% to 8.50%. Furthermore, both studies demonstrate that bricks containing GGBFS exhibit lower water absorption compared to control or conventional bricks without GGBFS. However, when the GGBFS content exceeded 25%, the bricks in the present study began to show a slight increase in water absorption. At a GGBFS content of 35%, water absorption increased to 9.60% for solid bricks and 9.90% for hollow bricks. This behavior is consistent with the findings from Surul et al. [26], which indicates that higher GGBFS contents resulted in either marginal improvements or reduced effectiveness in lowering water absorption. These results suggest that while moderate GGBFS replacement enhances the resistance of bricks to water ingress, excessive GGBFS content may limit further improvements in water absorption performance.

The water absorption results obtained in this study are consistent with the findings from [29], which reported that incorporating up to 25% GGBFS into brick mixtures leads to a reduction in water absorption. In the present study, bricks containing 25% GGBBS exhibited average water absorption values of 8.20% for solid bricks and 8.50% for hollow bricks. When the GGBFS content exceeded 25%, an increase in water absorption was observed. Specifically, bricks with 30% GGBFS showed a water absorption of 8.40%, while those with 35% GGBFS exhibited 9.60% absorption. Dom et al. [30] reported a similar trend and attributed the higher water absorption at elevated GGBFS contents to an increase in pore size within the brick matrix. Both studies concluded that bricks incorporating up to 25% GGBFS satisfy the requirements for Grade S classification, while bricks with GGBFS contents greater than 25% still meet the criteria for Grade A, indicating excellent resistance to water absorption.

This study also demonstrates that curing time significantly influences the water absorption of bricks. For solid bricks, the average water absorption decreased from 11.67% after 7 days of curing to 10.00% after 56 days. A similar trend was observed for hollow bricks, where water absorption reduced from 11.95% at 7 days to 10.30% at 56 days. These results indicate that prolonged curing enhances hydration and microstructural densification, thereby reducing the capacity of bricks to absorb water. In comparison, Hashim et al. [31] reported that incorporating 30% GGBFS into brick mixtures resulted in higher water absorption at 28 days when compared with non-GGBFS bricks; however, at higher replacement levels of 40% and 50% GGBFS, water absorption decreased with longer curing periods for both solid and hollow bricks. While in [31] an initial increase in water absorption for 30% GGBFS specimens was observed, the results of the present research show a more consistent reduction in water absorption across all curing periods. This contrast suggests that the interaction between GGBFS content and curing duration plays a critical role in governing water absorption behavior, with moderate GGBFS replacement and extended curing providing the best performance.

### 3.3. Results of Efflorescence Test 

At the end of 56 days of curing, efflorescence tests were conducted on five bricks from each design mix, and the results were evaluated in accordance with the BDS brick standard. Figure 5 shows the efflorescence test results at 56 days of curing. For both brick types examined, no efflorescence was observed in specimens containing 25% GGBFS, indicating optimal performance at this replacement level. For solid bricks, efflorescence levels progressively decreased as the GGBFS content increased, reaching a minimum value of 0% efflorescence at 25% GGBFS. Beyond this level, efflorescence began to increase again, with values of 4% and 3% recorded for bricks containing 30% and 35% GGBFS, respectively.

**Figure 6:**
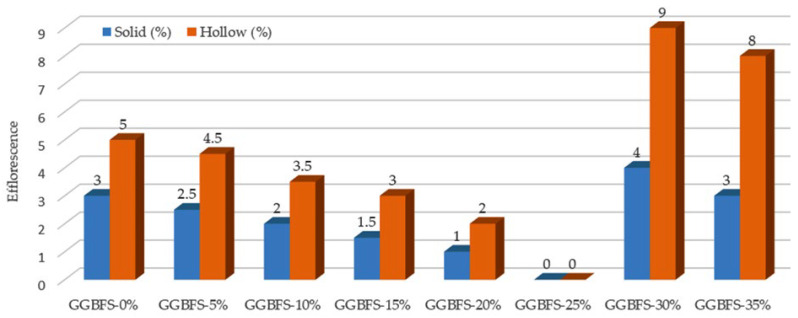
Efflorescence content (in %) test results of bricks at 56 days of curing.

A similar trend was observed for hollow bricks, where efflorescence levels steadily decreased up to 20–25% GGBFS, after which a sharp increase was noted. At 30% GGBFS, hollow bricks exhibited an efflorescence level of 9%, which slightly reduced to 8% at 35% GGBFS. Overall, the lowest efflorescence levels for both solid and hollow bricks were observed at a 25% GGBFS content. GGBFS contents exceeding this threshold resulted in increased efflorescence, with the effect being more pronounced in hollow bricks due to their higher porosity. Figure 6 shows the efflorescence test results at 56 days of curing.

A comparison between the efflorescence results obtained in this study and those reported in [33] indicates that the incorporation of GGBFS is effective in reducing efflorescence in clay bricks. Arya and Vanreyk [33] found that the greatest reduction in efflorescence occurred when 25% GGBFS was incorporated into the brick mix. This finding is strongly supported by our results. Here, for solid bricks, efflorescence decreased with increasing GGBFS content and reached 0% at 25% GGBFS. When the GGBFS content was higher, efflorescence reappeared, increasing to 4% at 30% GGBFS and then slightly decreasing to 3% at 35% GGBFS. In the case of hollow bricks, efflorescence levels were lowest up to approximately 20–25% GGBFS, after which a noticeable augmentation was observed (to 9% at 30% GGBFS and 8% at 35% GGBFS). Overall, these studies confirm that a 25% GGBFS replacement level produces the lowest efflorescence and therefore represents the most effective proportion for reducing the concentration of soluble alkaline salts within the brick matrix.

### 3.4. Results of Thermal Conductivity Test

**Figure 7:**
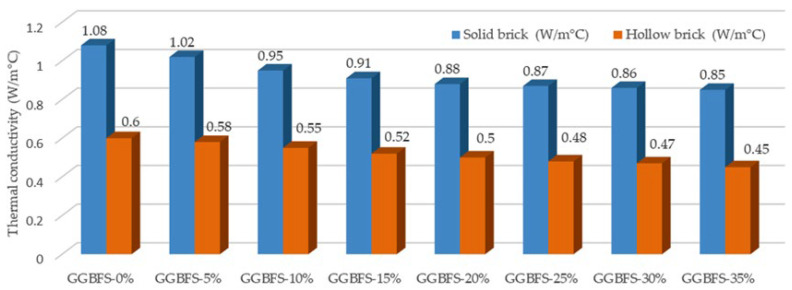
Thermal conductivity test results of bricks at 56 days of curing.

At 56 days of curing, thermal conductivity tests were carried out on five bricks from each design mix. The results indicate that the incorporation of GGBFS into both solid and hollow bricks leads to a reduction in thermal conductivity, thereby enhancing their insulating performance. For solid bricks, thermal conductivity decreased from 1.08 W/m·°C at 0% GGBFS to 0.85 W/m·°C at 35% GGBFS. A more pronounced reduction was observed for hollow bricks, where thermal conductivity decreased from 0.60 W/m·°C to 0.45 W/m·°C over the same GGBFS range. Figure 7 shows the thermal conductivity test results at 56 days of curing.

Across all mixtures, hollow bricks consistently exhibited significantly lower thermal conductivity than solid bricks. This behavior is attributed to the presence of internal air voids within hollow bricks, which act as natural thermal insulators and reduce heat transfer. Overall, the results demonstrate that GGBFS incorporation is beneficial in improving the thermal insulating characteristics of both types of bricks.

The findings of our research are consistent with those reported by Lopez-Perales et al. [32], who investigated the use of blast furnace slag (BFS) as a partial replacement for flint clay in refractory castables to enhance thermal resistance. They found that the incorporation of BFS produced a more homogeneous microstructure and reduced overall porosity, leading to improved thermal properties and increased heat resistance. A comparable effect was observed in the present study through the incorporation of GGBFS. Increasing the GGBFS content resulted in a consistent reduction in thermal conductivity across all brick samples. Notably, the hollow bricks exhibited a greater reduction in thermal conductivity compared to solid bricks. This behavior is likely attributable to the presence of internal air voids within the hollow bricks, which act as additional thermal barriers and further limit heat transfer. Such air voids were not considered in the BFS-based refractory castable examined by Lopez-Perales et al. [32]. To conclude, both studies provide strong evidence that the use of industrial by-product materials such as BFS and GGBFS can significantly influence the thermal performance of construction materials. By modifying the material composition and microstructural characteristics, these industrial wastes enhance thermal insulation and heat resistance, demonstrating their potential for use in sustainable and energy-efficient construction applications.

According to [33], the incorporation of GGBFS reduces the thermal conductivity of both solid and hollow bricks, thereby providing improved thermal insulation compared to conventional aggregates. In their study, the thermal conductivity of solid bricks decreased from 1.08 W/m·°C in bricks without GGBFS to 0.86 W/m·°C with a GGBFS content of 16%. Similarly, in the present study, the thermal conductivity of solid bricks decreased from 1.08 W/m·°C to 0.85 W/m·°C, although this reduction was achieved at a higher GGBFS replacement level of 35%. In contrast, Arya and Vanreyk [33] reported that the incorporation of 20% GGBFS, combined with laterite soil, reduced the thermal conductivity of hollow bricks from 1.08 W/m·°C to 0.76 W/m·°C. The results of our study indicate a more pronounced reduction for hollow bricks, where thermal conductivity decreased from 0.60 W/m·°C to 0.45 W/m·°C at a GGBFS content of 35%. These differences may be attributed to variations in material composition, brick geometry, and internal air voids presence, which play a significant role in enhancing thermal insulation performance.

The evaluation of mechanical and durability properties according to the BDS standard confirmed that bricks containing GGBFS exhibited good performance in terms of compressive strength, moisture absorption, and efflorescence, without requiring firing. The highest quality bricks (S-grade) were produced with 25% GGBFS, which also demonstrated the best water absorption and efflorescence results. All other GGBFS formulations achieved an A-grade, indicating suitability for masonry construction.

The use of GGBFS in brick production offers significant environmental benefits, including a reduced reliance on natural resources and a decreased need for kiln firing, which lowers energy consumption and emissions in mass production. While the study employed hand molding and hand compaction, it is suggested that machine compaction and optimized curing regimes could further enhance the mechanical and thermal performance of the bricks, facilitating their use in large-scale manufacturing.

### 3.5. Results on the Characteristic Compressive Strength

The characteristic compressive strength ($f_{k}$) results (Table 5) provide a conservative and structurally meaningful assessment of brick performance, as they incorporate both the mean strength and the statistical dispersion of the data.

Across all curing ages and brick types, a consistent and well-defined trend was observed with increasing GGBFS content. For solid bricks, $f_{k}$ increased progressively from 0% to 25% GGBFS at all curing ages. At 7 days, $f_{k}$ increased from 12.03 MPa (control) to 18.94 MPa at 25% replacement. This upward trend continued at 14 and 28 days, culminating at 56 days with a maximum characteristic strength of 23.63 MPa at 25% GGBFS, compared to 16.86 MPa for the control mix. This represents an improvement of approximately 40% in structural design strength.

A similar trend was observed for hollow bricks. At 56 days, $f_{k}$ increased from 14.28 MPa (0% GGBFS) to 18.63 MPa at 25% replacement, corresponding to an increase of approximately 30%. Although the absolute values were lower than those of solid bricks due to geometric effects, the proportional improvement with slag incorporation was consistent. Beyond 25% GGBFS, a reduction in $f_{k}$ was observed for both brick types at all curing ages. At 30% and 35% replacement levels, characteristic strength decreased, confirming the presence of a cement dilution effect when slag content becomes excessive.

The augmentation of $f_{k}$ with curing time demonstrates the sustained contribution of GGBFS to long-term strength development. While early-age (7-day) characteristic strengths showed notable improvement with increasing slag content, the most significant gains were observed at 56 days. For example, solid bricks with 25% GGBFS exhibited an increase in $f_{k}$ from 18.94 MPa at 7 days to 23.63 MPa at 56 days. This confirms that slag-containing mixtures benefit from continued hydration and secondary C–S–H formation, which enhances matrix densification and load-bearing capacity over time. The consistent increase in $f_{k}$ across curing ages supports the suitability of GGBFS-modified bricks for structural applications where long-term performance is critical.

The relatively small difference between the mean strength and $f_{k}$ across all mixes indicates limited statistical dispersion and high production consistency. We remark that the 20–25% GGBFS range not only produced the highest $f_{k}$ values but also demonstrated stable variability. This combination of high strength and controlled dispersion enhances structural reliability and reduces the probability of low-strength outliers. Given that structural design is governed by characteristic rather than mean strength, the improvement in $f_{k}$ is particularly significant. The approximately 30–40% increase in characteristic strength at optimal GGBFS content translates directly into increased load-bearing capacity or improved safety margins in masonry design.

Although hollow bricks consistently exhibited lower $f_{k}$ values than solid bricks, the effect of GGBFS replacement followed the same trend in both configurations. This confirms that slag primarily modifies the intrinsic mechanical properties of the cementitious matrix, while geometry influences the absolute magnitude of resistance. The proportional increase in characteristic strength with 25% GGBFS was comparable for both brick types, demonstrating that the beneficial effects of slag are material-driven and independent of unit configuration.

From a structural design perspective, the characteristic strength results clearly identify 25% GGBFS as the optimal replacement level under the present experimental conditions. This replacement level maximizes structural capacity while maintaining statistical reliability. The consistent increase in $f_{k}$ with the curing period further confirms that slag incorporation enhances long-term performance without compromising early-age strength. Conversely, replacement levels above 25% lead to a measurable reduction in characteristic strength, indicating that excessive slag content may adversely affect structural capacity.

The coefficient of variation (CV) obtained in this study ranged approximately between 4% and 27%, with most results falling within the 7–20% interval. These values are within acceptable limits for compressive strength testing of masonry units and indicate satisfactory experimental control and production consistency. Lower CV values were generally observed at later curing ages, particularly in the 20–25% GGBFS range, where variability decreased to as low as 7–10% for solid bricks at 56 days. This reduction in dispersion suggests improved microstructural homogeneity and more stable hydration products at optimal slag replacement levels.

Although some hollow brick specimens exhibited moderately higher CV values (approaching 25–27%), this can reasonably be attributed to geometric effects and stress concentration around the void, which naturally increase test sensitivity. Importantly, the overall magnitude of variability remains sufficiently low to justify the assumption of normal distribution used in the probabilistic calculation of characteristic strength. The relatively small difference between the mean strength and $f_{k}$ further confirms that dispersion did not significantly penalize the design values, thereby supporting the statistical reliability of the results.

Overall, the probabilistic evaluation of compressive strength confirms that moderate GGBFS incorporation significantly enhances the design strength of masonry units and provides improved reliability for structural applications [49].

## 4. Conclusions

This research examined the application of ground granulated blast furnace slag (GGBFS) as a partial replacement for natural sand in the production of sustainable, durable, and environmentally friendly sand bricks. Bricks were manufactured with GGBFS contents ranging from 0% to 35%, while maintaining a constant cement content of 15%. Different curing periods were applied before testing, and mechanical and physical properties were evaluated according to BDS specifications. The results indicate that GGBFS is a suitable alternative material that enhances brick performance while maintaining the required quality standards. However, it should be noted that laboratory results obtained under controlled conditions may not fully reflect long-term field performance under natural environmental exposures such as temperature fluctuations, moisture variations, freeze–thaw cycles, and chemical attack. Therefore, long-term durability should be further validated through extended field studies.

The key findings are:

⮚ Compressive Strength: After 56 days, solid bricks incorporating 25% GGBFS achieved the highest compressive strength, reaching Grade S (≥24 MPa). Hollow bricks with the same replacement level achieved Grade A (≥15 MPa), confirming their suitability for structural applications. When evaluated using characteristic compressive strength ($f_{k}$), which accounts for statistical variability, the 25% GGBFS mix also produced the highest design-relevant values, demonstrating an increase of approximately 30–40% compared to the control mix. This confirms that the strength improvement is not only reflected in mean values but is also statistically reliable for structural design purposes.
⮚ Statistical Reliability (CV and $f_{k}$): The coefficient of variation generally remained within acceptable limits for masonry testing, indicating consistent manufacturing and testing procedures. Lower variability was particularly observed in the 20–25% GGBFS range, where high characteristic strengths were achieved with controlled dispersion. The relatively small difference between the mean compressive strength and $f_{k}$ values confirm the reliability and structural safety of the optimized mixtures.
⮚ Water Absorption: An inverse relationship was observed between GGBFS content and water absorption. As the GGBFS percentage increased, water absorption decreased, with 25% GGBFS bricks exhibiting the lowest absorption (Grade S ≤ 10%). This indicates improved pore refinement and enhanced resistance to water penetration.
⮚ Efflorescence: Minimal efflorescence was observed in bricks containing 25% GGBFS. Higher replacement levels (30–35%) showed increased efflorescence formation, which may negatively affect aesthetic quality.
⮚ Thermal Conductivity: Thermal insulation performance improved with increasing GGBFS content. The thermal conductivity of solid bricks decreased from 1.08 W/m·°C to 0.85 W/m·°C, while hollow bricks decreased from 0.60 W/m·°C to 0.45 W/m·°C. The lower values in hollow bricks are attributed to the insulating effect of internal air voids.

Overall, the results demonstrate that incorporating 25% GGBFS provides the optimal balance between mechanical performance, durability-related properties, and statistical reliability. The improved characteristic compressive strength, combined with acceptable variability, confirms the suitability of this replacement level for structural masonry applications while contributing to more sustainable construction practices.

From a sustainability perspective, GGBFS usage reduces the use of non-renewable natural materials, such as sand and clay, by replacing them with alternative materials, thus decreasing GHG emissions produced by traditional brick production through kilns. GGBFS bricks can be produced more efficiently than traditional bricks due to the use of machine compaction instead of manual labor, and shorter curing times will also provide significant efficiency gains for producing GGBFS bricks at a larger scale. The mass production of GGBFS bricks represents a more sustainable solution for Bangladesh, where traditional brick production has led to many environmental challenges.

Future research should focus on validating the long-term performance of GGBFS-modified bricks under real environmental conditions. Field-based studies, including collaboration with construction companies, would allow in situ monitoring of mechanical performance, durability behavior, and service life under varying climatic conditions. Extended durability assessments should include freeze–thaw resistance, sulfate and chloride exposure, carbonation depth, and long-term moisture transport analysis. These investigations would provide a more comprehensive understanding of degradation mechanisms and structural reliability over time. In addition, future studies should explore the use of alternative industrial byproducts in combination with GGBFS to further enhance sustainability and promote circular economy practices in brick manufacturing. A detailed technical–economic, and life-cycle assessment is also recommended to evaluate the environmental and financial feasibility of large-scale production. Such an analysis would quantify potential reductions in carbon emissions, energy consumption, and raw material extraction. Finally, research should investigate the influence of automated or machine-compacted manufacturing processes on the mechanical, thermal, and durability performance of bricks. As production transitions from laboratory to industrial scale, understanding the interaction between manufacturing technology and material performance will be critical to maintaining quality while preserving environmental benefits.

## Figures and Tables

**Table 1:** Physical characteristics of Ordinary Portland Cement (OPC).

Properties	Test Results
Fineness	0.4 m^2^/kg
Density	1055 kg/m^3^
Specific gravity	2.95
Initial setting time	150 min
Final setting time	190 min
Consistency	27%
7 Days compressive strength	22.8 MPa
28 Days compressive strength	31.6 MPa

**Table 2:** Physical characteristics of ground granulated blast furnace slag (GGBFS).

Properties	Test Results
Fineness	0.312 m^2^/kg
Density	1020 kg/m^3^
Specific gravity	2.3
Moisture	0.8%

**Table 3:** Physical characteristics of the sand.

Properties	Test Results
Bulk density	1500 kg/m^3^
F.M modulus	1.20
Specific gravity	2.66
Absorption capacity	1.03%

**Table 4:** Mix design for cast samples with varying GGBFS contents.

Mix	Sand	GGBFS	Cement
Mix-1	85%	0%	15%
Mix-2	80%	5%	15%
Mix-3	75%	10%	15%
Mix-4	70%	15%	15%
Mix-5	65%	20%	15%
Mix-6	60%	25%	15%
Mix-7	55%	30%	15%
Mix-8	50%	35%	15%

**Table 5:** Characteristic compressive strength ($f_{k})$ and coefficients of variation (CV).

	GGBFS Content	$f_{k}$-7 d	$f_{k}$-14 d	$f_{k}$-28 d	$f_{k}$-56 d	CV-7 d	CV-14 d	CV-28 d	CV-56 d
Solid brick	0%	12.03	12.94	13.69	16.86	0.18	0.12	0.14	0.22
	5%	13.94	14.60	14.84	18.53	0.11	0.08	0.26	0.15
	10%	15.54	18.44	20.78	20.06	0.10	0.05	0.15	0.19
	15%	17.17	18.70	19.47	20.54	0.11	0.06	0.07	0.19
	20%	17.48	17.68	17.67	21.74	0.04	0.04	0.18	0.07
	25%	18.94	19.48	19.25	23.63	0.08	0.04	0.20	0.10
	30%	16.04	16.48	16.78	19.70	0.10	0.04	0.15	0.24
	35%	15.70	15.28	15.56	18.27	0.08	0.05	0.21	0.14
Hollow brick	0%	8.80	10.20	12.44	14.28	0.14	0.18	0.26	0.22
	5%	10.44	12.10	13.94	15.46	0.09	0.15	0.12	0.20
	10%	11.98	14.54	17.27	16.94	0.06	0.23	0.25	0.20
	15%	13.18	15.07	17.41	17.62	0.05	0.21	0.13	0.13
	20%	14.48	14.52	14.54	17.45	0.05	0.15	0.27	0.19
	25%	15.08	15.45	17.67	18.63	0.05	0.21	0.18	0.27
	30%	13.68	14.39	14.28	15.78	0.05	0.21	0.21	0.09
	35%	12.18	12.23	12.38	14.08	0.06	0.23	0.25	0.22

## Data Availability

The original contributions of this study are contained within the article. For further inquiries, please contact the corresponding author.

## Abbreviations

The following abbreviations are used in this manuscript:

A-class	High-Grade for Bricks
B-class	Medium-Grade for Bricks
BDS	Bangladesh Standard
BFS	Blast Furnace Slag
CBP	Clay Brick Powder
CFB	Circulating Fluidized Bed
CFBFA	Circulating Fluidized Bed Fly Ash
CO_2_	Carbon Dioxide
FA	Fly Ash
GGBFS	Ground Granulated Blast Furnace Slag
GHG	Greenhouse Gases
GO	Graphene Oxide
JHC	Joule Heating Curing
MPa	MegaPascals (unit of pressure)
OPC	Ordinary Portland Cement
PM_2.5_	Particulate Matter 2.5 (microns or smaller)
PM_10_	Particulate Matter 10 (microns or smaller)
S-class	Standard Grade for Bricks
SS	Steel Slag
W/C	Water/Cement Ratio
WBP	Waste Bamboo Powder
WCS	Waste Concrete Sludge
